# Epidemiology and Risk Factors of Rhegmatogenous Retinal Detachment: A Global and Historical Perspective

**DOI:** 10.3390/epidemiologia7040109

**Published:** 2026-08-13

**Authors:** Keshav Sehgal, Asterios Diafas, Nikolaos Dervenis, Panagiotis Dervenis

**Affiliations:** 1 Institute of Ophthalmology, University College London, London EC1V 9EL, UK; smgxkse@ucl.ac.uk; 2 Moorfields Eye Hospital, London EC1V 2PD, UK; asterios.diafas@nhs.net (A.D.); panagiotisdervenis@gmail.com (P.D.); 3 Institute of Ageing and Chronic Disease, University of Liverpool, Liverpool L7 8TX, UK

**Keywords:** rhegmatogenous retinal detachment, epidemiology, risk factors, prevalence, global incidence, historical perspective, myopia, retinal breaks

## Abstract

Background/Objectives: Rhegmatogenous retinal detachment (RRD) is a vision-threatening condition characterized by the separation of the neurosensory retina from the retinal pigment epithelium due to retinal breaks, leading to subretinal fluid accumulation. This narrative review aims to provide a comprehensive overview of the epidemiology, risk factors, and historical evolution of RRD from a global perspective, highlighting trends, regional variations, and key advancements to inform clinical practice and future research. Methods: A targeted literature search was conducted using databases such as PubMed and Scopus to identify studies published from 1970 to 2025 on RRD epidemiology, risk factors, incidence rates and temporal trends. The inclusion criteria focused on population-based studies, meta-analyses, and reviews. The findings were synthesized narratively, with quantitative estimates reported where available from the included studies and meta-analyses. Results: The global annual incidence of RRD is estimated at 12.17 per 100,000 people, with significant regional variations: it is the highest in Europe (14.52 per 100,000) and lower in the Americas (8.95 per 100,000). The incidence of rhegmatogenous retinal detachment has risen by 5.4 cases per 100,000 people per decade, with projections suggesting that it could double over the next 20 years. The key risk factors include myopia (3–39-fold increased risk depending on severity), age (peak in 60–70s), male sex, cataract surgery, and trauma. Conclusions: The RRD incidence is rising globally, driven by aging populations and increasing myopia prevalence, with myopia as the strongest potentially modifiable risk factor. Historical advancements underscore the importance of early detection and surgical intervention. Future efforts should focus on applying preventive strategies in high-risk groups and addressing regional disparities in access to care.

## 1. Introduction

Rhegmatogenous retinal detachment (RRD) represents a critical ophthalmic emergency that, if untreated, can lead to permanent vision loss. It occurs when liquefied vitreous enters through a full-thickness retinal break, separating the neurosensory retina from the underlying retinal pigment epithelium. Globally, RRD contributes significantly to visual morbidity, particularly among older adults. Its significance lies in its potential for progression (occasionally rapid) and high morbidity, affecting quality of life and imposing substantial healthcare burdens worldwide [1,2].

The historical trajectory of RRD management reveals a remarkable transformation. RRD was considered incurable until the early 20th century, with blindness as the inevitable outcome. Key milestones, such as Jules Gonin’s recognition of retinal tears as the causative factor in 1919, revolutionized its understanding and treatment [3].

Epidemiologically, RRD exhibits considerable variability across populations and geographies. The global incidence varies, influenced by demographic factors like age, sex, and refractive errors, with emerging trends showing an increase over time. Risk factors such as myopia, trauma, and prior ocular surgery further modulate the susceptibility [1,2,3].

This review aims to provide a thorough synthesis of the epidemiology and risk factors of RRD, viewed through a global and historical prism. By integrating data from diverse sources, including meta-analyses and longitudinal studies, we seek to illuminate regional disparities, temporal shifts, and evolving understandings. Controversies, such as the role of prophylactic treatments in high-risk eyes or the impact of socioeconomic factors on the outcomes, will be addressed where relevant. The ultimate goal is to highlight areas for intervention, foster awareness among clinicians and policymakers, and stimulate research into preventive modalities.

## 2. Materials and Methods

To compile this review, a rigorous and structured approach was adopted to ensure comprehensive coverage and minimize bias.

### 2.1. Search Strategy

A structured literature search was initiated in major academic databases: PubMed and Scopus. The timeframe was set from 1 January 1970 to 21 September 2025, to capture both foundational historical works and the most recent epidemiological data [4]. The search method was constructed using Boolean operators and included combinations such as (“rhegmatogenous retinal detachment” OR “RRD”) AND (“epidemiology” OR “incidence” OR “prevalence” OR “risk factors” OR “global” OR “historical” OR “temporal trends” OR “regional variations”). Truncation and wildcard symbols were employed to broaden the search, e.g., “detach*” for detachment-related terms [2].

### 2.2. Scope of Included Literature

The inclusion criteria were stringent: only peer-reviewed articles in English were considered, with a focus on systematic reviews, meta-analyses, population-based cohort studies, and large-scale registries that provided robust data on the incidence, risk factors, or historical aspects [4]. The studies had to report on primary RRD cases, excluding tractional or exudative detachments. For historical sections, seminal papers and review articles detailing key milestones were prioritized [5].

The exclusion criteria encompassed case reports, small series analyses, animal or in vitro studies, and publications lacking methodological rigor, such as those without clear definitions of RRD or inadequate statistical controls [2].

### 2.3. Study Selection

The titles and abstracts identified through the database search were reviewed for relevance to the objectives of this narrative review. The full texts were then assessed for eligibility on the basis of the study design, relevance to RRD epidemiology or risk factors, and methodological clarity.

### 2.4. Evidence Synthesis

Key information from the included studies was recorded using a structured template, capturing variables like the study design, population demographics, incidence/prevalence rates (with confidence intervals), odds ratios for risk factors, temporal trends, and historical contributions [6]. For quantitative synthesis, meta-analytic estimates from the included reviews were adopted when the heterogeneity (as measured by the I^2^ statistics) was low to moderate; otherwise, narrative synthesis was used [4]. No primary data collection occurred, and all results were based on previously published findings. Where data gaps existed, such as in understudied regions like Africa, estimates were noted as provisional and contextualized with potential biases like underreporting [7].

### 2.5. Use of Artificial Intelligence

Regarding the use of generative artificial intelligence (ChatGPT-5), it was used solely during the preliminary stages of manuscript preparation to assist with literature screening and keyword refinement. Specifically, GenAI assisted in summarizing large volumes of abstracts. However, it was not used for study selection decisions, data extraction or the interpretation of findings. All the evidence included in the review, together with the final manuscript text and conclusions, was critically reviewed and verified by the authors.

## 3. Results

The results are organized into subsections addressing the historical evolution, global epidemiology with temporal trends, and detailed risk factors of RRD. Synthesis was performed where applicable from the studies identified, providing a multifaceted view.

### 3.1. Historical Perspective

The historical narrative of RRD reflects a progression from mystery to mastery in ophthalmic science. In the 19th century and early 20th century, retinal detachment was poorly understood, often conflated with other ocular pathologies. Pioneers like Albrecht von Graefe in the 1850s described detachment, but attributed it to choroidal effusions or hypotony, lacking insight into the vitreous–retina interface [8]. Treatment attempts were rudimentary and ineffective, including bed rest, pressure bandages, or even trephination, yielding dismal outcomes with blindness rates approaching 100% [7].

The understanding of RRD has evolved dramatically over the past century. A paradigm shift occurred in 1919 when Jules Gonin, through meticulous clinicopathological correlations, pinpointed retinal tears as the etiological cornerstone of RRD [9]. His ignipuncture technique—using a heated probe to cauterize tears and promote chorioretinal adhesion—achieved reattachment in about 50% of cases, a revolutionary success [5]. This method, detailed in Gonin’s 1934 monograph, laid the foundation for modern vitreoretinal surgery [9]. The 1930s brought refinements, with Björn Rosengren introducing diathermy for more precise coagulation and air injection into the vitreous to tamponade the retina, improving the rates to 60–70% [10].

The post-war era accelerated innovation. In the 1950s, Charles Schepens and Ernst Custodis developed scleral buckling, a technique involving external scleral indentation with silicone or polyethylene materials to counteract vitreous traction and close breaks [11]. This approach, combined with cryotherapy for adhesion, elevated the success to 70–80% and became the gold standard for uncomplicated RRD [12]. Schepens’ indirect ophthalmoscope further enhanced the visualization of the peripheral retina, aiding in diagnoses and treatment [13].

The 1970s ushered in the vitrectomy era with Robert Machemer’s invention of pars plana vitrectomy (PPV) in 1971 [14]. This internal approach allowed for the removal of pathological vitreous, the relief of traction, and the use of tamponade agents like silicone oil or gas, proving invaluable for complex cases involving proliferative vitreoretinopathy (PVR) [15]. The success rates climbed to 85–90%. The 1980s saw pneumatic retinopexy by Hilton and Grizzard, an office-based procedure using intravitreal gas bubbles and laser/cryo for superior breaks, offering minimally invasive options with 70–80% success in selected patients [16].

Into the 21st century, the advancements included microincision vitrectomy surgery (MIVS) with 23–27 gauge instruments, reducing postoperative inflammation and the recovery time [17]. Endolaser, wide-field viewing systems, and heavy tamponades have further refined the outcomes, achieving over 95% anatomical success in primary cases [18]. Despite these strides, challenges persist in preventing recurrence and managing PVR, which complicates 5–10% of cases [19]. Today, the surgical success exceeds 90%, with ongoing refinements in microsurgical techniques [10,20,21,22]. The key historical milestones in RRD management are summarized in Table 1.

### 3.2. Global Epidemiology and Temporal Trends

Epidemiological data reveal RRD as a condition with a substantial global burden, affecting approximately 1 in 10,000 people annually. A 2019 meta-analysis of five studies estimated a pooled incidence of 13.3 per 100,000 (95% CI: 11.3–15.6), translating to over 100,000 new cases yearly worldwide [4]. A more recent meta-analysis of 33 studies from 21 countries estimated the RRD incidence to be 12.17 (95% CI: 10.51–14.09), being highest in Europe (14.52, 95% CI: 11.79–17.88) [1]. Regional heterogeneity is pronounced; in Europe, this is driven by aging demographics and cataract surgery increases in countries like Germany (24.8/100,000) and Denmark (20.72/100,000) [23,24,25]. The Western Pacific region follows, with an incidence of 10.39 per 100,000 (95% CI: 10.26–10.52) in Korea and 10.4 per 100,000 in Japan amid the myopia epidemic [26,27].

In the Americas, the incidence is lower, at 8.95 per 100,000 (95% CI: 6.73–11.92), with US studies showing 12–18 per 100,000 in specific cohorts, and Brazil at around 7–9 per 100,000 [28,29]. African data are sparse, with estimates of 7–10 per 100,000 in South Africa, likely underestimated due to limited surveillance and access to care [1,7]. In Asia, the variations are stark: China’s rates range from 7.26 in rural areas to 26.2 in urban high-myopia zones, while India reports 3–5 per 100,000, possibly reflecting diagnostic gaps [18,30].

Temporal trends demonstrate a consistent increase, with a meta-analysis indicating a 5.4 per 100,000 rise per decade from 1997 to 2019 [4]. In The Netherlands, the incidence grew from 7.0 in 1971–1981 to 18.2 per 100,000 by 2010 [6]. Germany’s Gutenberg Health Study noted a jump to 24 per 100,000 by 2021 [31]. Projections suggest doubling by 2040, attributed to population aging (global elderly proportion rising from 10% to 16% by 2050) and a myopia surge (affecting 50% of the world by 2050) [32]. Seasonal patterns show summer peaks, correlated with higher temperatures, dehydration, or outdoor activities increasing the trauma risk [33,34,35]. Ethnic differences include higher rates in Caucasians versus Asians or Blacks, though myopia adjusts this in Asian populations [36]. The incidence rates by region are outlined in Table 2.

The prevalence of RRD, while less frequently reported than the incidence due to its acute nature, varies globally and reflects the cumulative disease burden in populations. Studies estimate a point prevalence ranging from 0.05% to 0.1% in general populations, with higher rates in high-risk groups such as those with severe myopia or prior ocular surgery [2,38]. In regions with robust healthcare systems, such as Scandinavia, the prevalence is better documented due to comprehensive registries, reaching approximately 0.08% in Denmark [24]. In contrast, underreporting in low-resource settings, such as parts of Africa, likely underestimates the true prevalence, potentially masking a significant public health challenge [7].

### 3.3. Risk Factors

The risk factors for RRD are multifactorial, encompassing ocular, demographic, and environmental elements. Myopia is paramount, with mild myopia (<3 diopters) conferring a 3-fold risk and high myopia (>6 diopters) up to 39-fold, due to axial lengthening causing peripheral retina degeneration and vitreous syneresis. Population studies in Asia, where myopia affects 80–90% of young adults, have linked this to 50–70% of RRD cases [10,39,40,41,42,43,44].

Age is a non-modifiable factor, with the incidence peaking at 60–70 years owing to PVD, occurring in 50–60% of individuals over 60. Pre-PVD, younger myopes (20–40 years) form a bimodal peak [45]. Male sex elevates the risk 1.5–2-fold, attributed to higher trauma and occupational exposures; globally, males comprise 55–65% of cases [4,46].

Ocular surgery, especially phacoemulsification cataract extraction, increases the risk 4–10-fold postoperatively, with the cumulative incidence reaching 1–2% within 5–10 years, due to altered vitreous dynamics [6,36]. Trauma accounts for 10–15% of cases, with blunt injuries causing posterior vitreous detachment, retinal dialysis, commotio retinae and tears; the risks are variable, but up to 10-fold in contact sports [47,48].

Genetic factors include vitreoretinopathies like Stickler syndrome (COL2A1/COL11A1 mutations), which is associated with a high lifetime risk of RRD; bilateral involvement is common, particularly in type 1 disease [40,49,50]. Lattice degeneration, present in 6–8% of the population, raises the risk 20-fold [51]. Other associations include: diabetes (mild increase via vascular changes) [52], smoking (1.5-fold via oxidative stress) [53], and a low socioeconomic status (via delayed care) [33].

In pediatrics, RRD is rare (0.38–0.69 per 100,000) and is often linked to trauma (40%), myopia (30%), or congenital anomalies (15%), with mental retardation elevating the rates to 11% due to self-injury [54]. The key risk factors and related risks are summarized in Table 3.

Prognostic factors significantly influence the RRD outcomes, with timely intervention being paramount. Macular involvement at presentation worsens the visual prognosis, with studies showing only 40–60% of patients achieving 20/40 vision post-surgery if the macula is detached [58]. The duration of detachment before repair also impacts recovery, with delays beyond 7 days reducing the functional outcomes [59]. Proliferative vitreoretinopathy (PVR), occurring in 5–10% of cases, is a major predictor of surgical failure, increasing the re-detachment risk [60]. A younger age and the absence of high myopia correlate with better anatomical success [45].

## 4. Discussion

The synthesized findings underscore rhegmatogenous retinal detachment (RRD) as an escalating public health concern, with the incidence trends mirroring broader societal shifts such as population aging and the myopia crisis [4,23]. In Europe and North America, where the life expectancy exceeds 80 years, the age-related peak in the 60–70s drives higher rates, compounded by widespread cataract surgeries—over 20 million annually worldwide [36]. In contrast, Asia’s surge is myopia-centric, with urbanization and near work activities (e.g., screen time) fueling a prevalence increase from 20% in the 1970s to 80% today among youth, projecting millions at an elevated RRD risk [32,61].

Historical advancements illustrate how scientific insight translates to clinical gains: from Gonin’s 50% success to the modern >95%, enormous progress in the management of RRD has been achieved [9,18]. Yet, disparities endure—developing regions like Africa report lower incidences, likely artifactual from underdiagnosis, where access to vitreoretinal specialists is scant (e.g., 1 per million in sub-Saharan Africa vs. 1 per 100,000 in the US) [7,53]. Socioeconomic factors exacerbate this: low income groups delay presentation, increasing proliferative vitreoretinopathy complications and poor outcomes [33].

The risk factor interplay is complex; myopia synergizes with age and surgery, while genetics modulate in 5–10% of cases [38,50]. The role of prophylactic retinopexy for asymptomatic retinal breaks, lattice degeneration or fellow eyes after primary RRD remains controversial. Although treatment may be considered in selected high-risk eyes, many lesions do not progress to RRD, and any potential benefit must be balanced against limited supporting evidence and the risk of unnecessary intervention and resource use [62]. Refractive lens exchange (RLE), which is increasingly being performed for presbyopia and high refractive errors, elevates the RRD risk similarly to cataract surgery due to altered vitreous dynamics [58]. Studies indicate a 3–8-fold increased risk post-RLE, particularly in myopic patients, with a cumulative incidence of 1–3% within 5 years [57]. This underscores the need for preoperative risk assessment and patient counseling, especially in younger myopes where RLE is elective, to balance the visual benefits against potential retinal complications [38].

The limitations of this review include the reliance on heterogeneous studies, with varying definitions and ascertainment biases. The underrepresentation of low-resource areas hampers global generalizability. Future directions could include: longitudinal cohorts in underrepresented regions, genome-wide studies for polygenic risks, and interventions like myopia control (atropine drops, orthokeratology) potentially halving the RRD incidence [63,64]. AI for predictive modeling and telemedicine for early screening could also improve care in underserved areas [65]. Ultimately, integrating epidemiology with policy—e.g., school-based myopia prevention—offers promise for burden reduction [63].

## 5. Conclusions

In summary, RRD’s global incidence is on an upward trajectory, propelled by aging demographics, an increase in cataract surgery and the myopia epidemic, with myopia standing out as the foremost modifiable risk. Historical innovations have transformed the prognosis, yet regional inequities in access and treatment persist. Prioritizing prevention in vulnerable groups and enhancing surveillance will be pivotal in alleviating the future impact of this vision-threatening disorder.

## Figures and Tables

**Table 1:** Historical milestones in the understanding and treatment of rhegmatogenous retinal detachment.

Year	Milestone	Contributor	Description
1919	Recognition of retinal breaks	Jules Gonin	Introduced ignipuncture for sealing tears.
1930s	Diathermy and air injection	Rosengren	Early methods to reattach retina.
1950s	Scleral buckling	Schepens, Custodis	Indentation to relieve traction.
1971	Pars plana vitrectomy	Machemer	Vitreous removal for complex RRD.
1986	Pneumatic retinopexy	Hilton, Grizzard	Gas bubble tamponade.
2000s+	Microincision vitrectomy	Various	Smaller gauges for reduced morbidity.

**Table 2:** Global incidence rates of rhegmatogenous retinal detachment by region (per 100,000 people).

Region	Incidence Rate (95% CI)	Key Studies/Countries	Notes
Europe	14.52 (11.79–17.88)	UK, Germany, Denmark	Highest global rates; increasing trend [1,25].
Western Pacific	10.55 (8.71–12.75)	Japan, Korea, Australia	Rising in Asia due to myopia epidemic [26,27,37].
Americas	8.95 (6.73–11.92)	USA, Brazil	Lower, but increasing; ethnic variations noted [28,29].
Africa/Middle East	7–10 (estimated)	Limited data; e.g., South Africa	Underreporting likely; lower than global average [7].
Global Average	12.17 (10.51–14.09)	Meta-analysis of 33 studies	Increasing at 5.4/decade [1].

**Table 3:** Key risk factors for rhegmatogenous retinal detachment and associated relative risks.

Risk Factor	Relative Risk/Odds Ratio	Evidence Level	Notes
Myopia (mild, <3D)	3-fold	High (meta-analysis)	Axial elongation thins retina [10,39].
High myopia (>6D)	10–39-fold	High	Strongest modifiable factor [39,40].
Age (>60 years)	2–5-fold	Moderate	Linked to vitreous detachment [10].
Male sex	1.5–2-fold	High	Higher trauma rates [41,55].
Cataract surgery	4–10-fold post-op	High	Increases vitreous mobility/detachment [42,56].
Trauma	Variable (up to 10-fold)	Moderate	Direct cause in 10–15% of cases [43].
RLE	3–8-fold	Low–moderate	Particularly relevant in myopic patients [57].
Genetic (e.g., Stickler)	10–20% bilateral risk	Low–moderate	Hereditary vitreoretinopathies [40].

## Data Availability

Data supporting the reported results are available from the cited references.
